# P05.73. A feasibility study of caregiver-provided massage for comfort care in Veterans with cancer

**DOI:** 10.1186/1472-6882-12-S1-P433

**Published:** 2012-06-12

**Authors:** L Kozak, E Vig, C Simons, E Eugenio, M Chapko

**Affiliations:** 1 HSR&D, VA Puget Sound Health Care System & UW School of Public Health, Seattle, USA; 2 VA Puget Sound Health Care System, Seattle, USA

## Purpose

The purpose of this study was to assess the feasibility of using a multimedia program to instruct caregivers in providing massage as supportive care for Veterans with cancer.

## Methods

Dyads received a training program including DVD and manual, and were instructed to watch and practice at least 20 minutes/day over 8 weeks. The training program was specially developed for caregivers of cancer patients and previously tested in a randomized trial with non-veteran population. Feasibility assessed partner availability, perceived burden, clarity of instructional materials and compliance with training materials, weekly massage practice, and returning of data collection instruments.

## Results

26 patient-caregiver dyads (92% male patients and 95% female caregivers) were recruited; 15% wanted to participate but did not have a partner. Only 11/26 pairs, all of which were spouse dyads, completed the study, 38.5% of 26 recruited were unable to complete study because of being overwhelmed with caregiving activities. All patients were undergoing chemotherapy, radiation or both. For the 71.5% who completed 8 weeks, compliance with training materials was high (Mean times reading manual over 8 weeks=15.83). Dyads reported high satisfaction with training, 91% declared training was very clear and easy to follow. Compliance with weekly practice was also high (Mean times caregiver provided massage over 8 weeks= 43.83). Compliance with data collection and returning of instruments was acceptable for caregivers but poor for patients, who perceived forms as a burden.

## Conclusion

Massage has been shown to support co-management of pain and anxiety in palliative care. Although massage is widely used at cancer centers around US, the VA system lacks occupational codes to hire LMPs to offer massage. This study showed feasibility of training caregivers of Veterans to provide simple massage for comfort care at home. Future studies should include a larger population and consider provision of caregiver support to facilitate adherence.

